# Can Daily Dietary Choices Have a Cardioprotective Effect? Food Compounds in the Prevention and Treatment of Cardiometabolic Diseases

**DOI:** 10.3390/metabo14060296

**Published:** 2024-05-23

**Authors:** Elżbieta Szczepańska, Barbara Janota, Marika Wlazło, Magdalena Gacal

**Affiliations:** 1 Department of Human Nutrition, Department of Dietetics, Faculty of Public Health in Bytom, Medical University of Silesia in Katowice, Jordana 19 Street, 41-808 Zabrze, Poland; 2 Department of Basic Medical Sciences, Faculty of Public Health in Bytom, Doctoral School of Medical University of Silesia in Katowice, Piekarska 18 Street, 41-902 Bytom, Poland; 3 Faculty of Public Health in Bytom, Doctoral School of Medical University of Silesia in Katowice, Piekarska 18 Street, 41-902 Bytom, Poland

**Keywords:** metabolic syndrome, cardiovascular disease, diet, nutrients, food

## Abstract

Cardiovascular diseases accompanying metabolic syndrome comprise one of the leading causes of death worldwide. The medical community undertakes attempts to improve treatment options and minimize cardiovascular diseases’ numerous consequences and exacerbations. In parallel with pharmacotherapies provided by physicians, nutritionists are developing strategies for diet therapy and prevention based on lifestyle changes, with high success rates. Consumption of specified food compounds included in various products with proven protective properties can be helpful in this regard. Due to the wide possibilities of diet in metabolic health promotion, it seems necessary to systematize information about the metabolically protective and cardioprotective properties of fiber, probiotic bacteria, plant sterols, folic acid, vitamins B12, C, and E, PUFAs, lycopene, polyphenols, arginine, CoQ10, and allicin. The aim of this review was to present the food compounds with potential use in cardiometabolic prevention and diet therapy based on the latest available literature.

## 1. Introduction

According to 2020–2021 estimates, cardiovascular diseases (CVDs) comprise one of the leading causes of death globally [1,2]. Cardiovascular disorders accompany metabolic syndrome, which is diagnosed when three of the following five occur: increased glucose levels, elevated triglyceride concentration, hypertension, low high-density lipoprotein concentration, and obesity [3]. According to a meta-analysis involving data from 16,245 patients, individuals with low HDL cholesterol levels are particularly at risk for cardiovascular complications, underscoring the critical need for preventive measures in those with diagnosed lipid disorders [4]. Zalewska et al. demonstrated that individuals with metabolic syndrome exhibit low levels of catestatin, a peptide that positively influences glucose tolerance. Its levels correlate positively with HDL-c levels and negatively with body mass index and the 10-year risk of atherosclerotic cardiovascular disease. The presence of metabolic syndrome itself is associated with an increased risk of atrial fibrillation, coronary artery disease, hypertension, and stroke [5,6]. Additionally, its presence heightens the risk of death due to cardiovascular diseases [7]. The interdependence between cardiovascular diseases and metabolic diseases requires particularly broad preventive and therapeutic interventions. 

The medical community is constantly engaged in improving treatment options and minimizing the numerous adverse effects resulting from chronic cardiac diseases and their exacerbations. In parallel with the drug therapies provided by physicians, nutritionists are developing strategies for diet therapy and prevention based on lifestyle changes, with high success rates [8,9]. 

Both prevention and dietary therapy of metabolic and cardiovascular disorders includes, among others, the consumption of products containing polyunsaturated fatty acids, phytochemicals (stanols, sterols), fiber, B vitamins, and vitamins C and E, as well as natural substances with free-radical-scavenging activity, probiotics, and products from which selected ingredients, such as salt or simple sugars, have been intentionally eliminated [10]. The use of foods which will provide many bioactive compounds in prevention and dietary treatment does not force a chosen dietary model but encourages the daily consumption of various products with proven protective properties for the body. With the assistance of dietitians, selecting the most beneficial nutritional strategy to implement recommendations can prove to be an effective method for preventing and supporting the treatment of metabolic diseases and their complications [11]. It seems necessary to systematize the newest data about diet prevention and diet therapy possibilities in cardiometabolic disorders so that they may be easily used by doctors, nutritionists, and patients. The aim of this review was to present food ingredients and their potential use in the prevention and treatment of cardiometabolic diseases, based on the latest available literature.

## 2. Methods

This literature review used the PubMed database (July 2023–May 2024) and included both Polish- and English-language publications. The following keywords were used in the search: cardiometabolic disorders and diet (PubMed 1668 publications); metabolic syndrome and CVD (PubMed 1056 publications); CVD and diet (PubMed 2168 publications); metabolic syndrome and diet (PubMed 5164 publications). This paper was based on 113 publications. The literature included experimental studies, meta-analyses, cross-sectional analyses, and reviews. The last search was performed on 18 May 2024. 

## 3. Food Compounds in Cardiometabolic Prevention 

### 3.1. Plant Sterols and Stanols

Plant sterols, which are derived from vegetable oils, bread, vegetables, cereals, nuts, and fat spreads, structurally and functionally resemble cholesterol. Their average daily intake in the usual diet mostly does not exceed 500 mg [12]. A slightly higher proportion of compounds can be found in the diet of vegetarians, where it can be up to 600 mg [13]. A potential mechanism by which plant sterols and stanols contribute to cholesterol reduction and thus lower the cardiovascular risk is based on inhibiting its intestinal absorption by creating competition for the solubilization phase. Transintestinal cholesterol excretion (TICE) is another mechanism by which plant sterols and stanols contribute to cholesterol homeostasis. This action is mainly based on the prevention of cholesterol ester translocation and esterification, prevention of de novo cholesterol synthesis through reducing hydroxymethylglutaryl-CoA reductase, expression of sterol reductase C24, and incorporation of cholesterol esters into chylomicrons. Additionally, plant sterols, by mediating the regulation of hepatocytic bile acid synthesis, regulate the secretion of very-low-density lipoproteins (VLDLs) and increase cholesterol excretion via the nonbiliary TICE pathway [14,15,16]. The action of the mentioned substances in terms of lowering LDL cholesterol fraction is also significant due to their role in counteracting atherosclerosis occurring in metabolic syndrome. Low-density lipoproteins have the ability to penetrate the arterial endothelium where, under the influence of proteoglycans, free radicals, and macrophages, they contribute to the formation of atherosclerotic plaque [17].

However, it is worth pointing out the relationship between the effect of phytosterols on the lipid profile and the regularity of their use. Research indicates a cholesterol-lowering effect noticeable after 3 weeks of routine use. It also seems to be important to consume food containing phytosterols after one of the main meals, such as lunch or dinner [18]. Previous studies confirm the efficacy of 2 g plant sterol supplementation in lowering cholesterol, while questioning the beneficial effect of sterols and stanols from natural dietary sources on LDL-C levels. However, it is noteworthy that despite studies indicating a mild cholesterol-lowering effect in patients supplementing plant sterols with diet, their elimination from daily consumption is associated with an increase in LDL-C [13]. Furthermore, the maximum safe daily dose of plant sterols has not been determined, and researchers suggest the need to identify possible adverse effects and establish the duration for which they can be used [19].

### 3.2. Omega-3 Fatty Acids

Current research and expert opinions point to the colossal role of dietary fatty acids in the pathogenesis and treatment of lipid disorders, as well as in improving tissue sensitivity to insulin through their influence on the expression of genes responsible for glucose metabolism [20,21]. These antilipemic effects are attributed to the multidirectional mechanism of action of polyunsaturated fatty acids and include the following:

- Positive impact on the structure and function of cell membranes through the positive effects of EPA on the thickness of cell membranes, increasing their fluidity and inhibiting the formation of cholesterol domains;
- Reduction of inflammation that significantly contributes to atherosclerosis by EPA creating a competition for arachidonic acid in metabolic changes, leading to less inflammatory and chemotactic metabolic products;
- Lowering blood pressure, with this effect being significantly more likely to be seen in hypertensive patients. The mechanism is based on reducing the activity of angiotensin-converting enzyme and the vagus nerve, improved vasodilatory response and arterial wall compliance;
- Antithrombotic and anti-inflammatory effects that reduce the risk of thrombosis responsible for stroke and myocardial infarction by reducing thromboxane A2 production in favor of increased synthesis of thromboxane A3;
- Improved cardiac contractility by stabilizing increased myocyte activity;
- Beneficial effects on the lipid profile by lowering triglyceride levels through a mechanism involving the reduction of hepatic lipogenesis [22].

**Figure 1:**
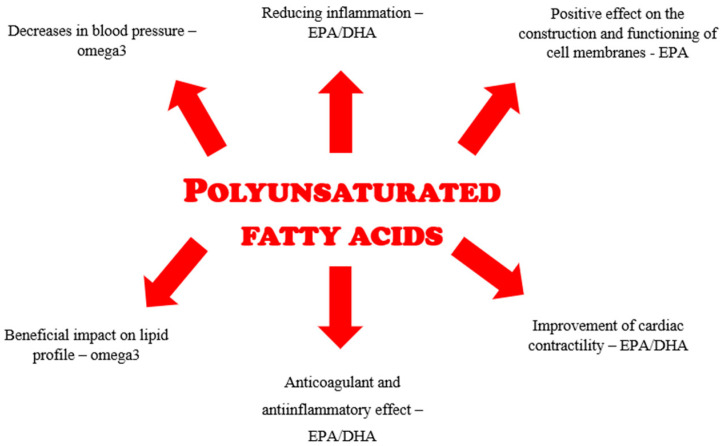
Functions of polyunsaturated fatty acids in terms of cardiometabolic risk reduction.

Figure 1 shows the functions of individual polyunsaturated fatty acids in terms of CVD risk reduction.

Studies on reducing the risk of death from cardiovascular incidents and stroke primarily indicate the benefits of consuming adequate amounts of fish (≥2 times/week) as a good source of polyunsaturated fatty acids. The highest content of polyunsaturated fatty acids is characterized by salmon, tuna, mackerel, and trout [11]. Alpha-linolenic acid, found in edible chestnuts, seed-derived oils, and some vegetables, lowers blood triglycerides and reduces postprandial lipemia [23]. To reduce the risk of mortality from coronary artery disease and cardiovascular diseases, it is recommended that the intake of PUFAs be 250–500 mg/day and ALA be 0.6–1.2% of energy intake [24]. Due to their content of monounsaturated and polyunsaturated fatty acids, olive oil, and canola oil show cardioprotective effects [25]. Current studies confirm that dietary enrichment with extra-virgin olive oil reduces the risk of cardiovascular events in the high-risk group by 30%. However, it is worth noting the multifaceted approach to diet in these studies [26].

Lambert et al. examined the effect of supplementation with phytosterols and omega-3 PUFAs on lipid metabolism parameters in overweight and first-degree obese people. It was observed that consuming milk with the addition of docosahexaenoic acid (DHA) and eicosapentaenoic acid (EPA) for 28 days resulted in a statistically significant reduction in the concentration of triglycerides and very-low-density lipoprotein. Therefore, it can be concluded that supplementation with n-3 PUFAs and phytosterols brings beneficial effects in the fight against lipid disorders [15]. Furthermore, current studies indicate a beneficial interaction between vitamin B12 and omega-3 PUFAs. Conducted analyses confirm that the total amount of vitamin B12 is positively associated with the plasma concentration of omega-3 PUFAs [27].

However, it is noteworthy that polyunsaturated fatty acids in products and supplements can oxidize quickly, potentially resulting in the delivery of substances with pro-inflammatory properties. Scientists emphasize the need to raise public awareness about the potential risks associated with the intake of these fatty acids [28].

### 3.3. Coenzyme Q10

Coenzyme Q10 (CoQ10) is an essential ingredient for electron transport in the respiratory chain that is part of the processes responsible for producing energy in the body. Furthermore, CoQ10 is an anti-inflammatory component that stabilizes cardiac calcium channel function, lowers total cholesterol and LDL fraction, and reduces the risk of atrial fibrillation [29,30]. A decrease in CoQ10 levels has been observed in CVD, and it predisposes one to increased heart failure [30,31]. There are reports on the benefits of CoQ10 supplementation, especially among adult people on statins, which prevent the endogenous synthesis of the compound. According to available studies, CoQ10 supplementation at a level of 400–500 mg per day has a significantly beneficial effect on total cholesterol [32]. However, these findings are limited, prompting the need for further research and evaluation of the necessity for additional CoQ10 intake beyond the standard diet. Products that can be considered cardiometabolic-protective due to their CoQ10 content include, among others, wheat germ, fatty fish, oils, and nuts [33]. It is worth bearing in mind that in addition to CoQ10, these products contain multiple other components, such as vitamin E, plant sterols, and unsaturated fatty acids, the supply of which brings health benefits.

### 3.4. Dietary Fiber

Dietary fiber is recommended for people with hypertension, coronary artery disease (CAD), myocardial infarction (MI), hypercholesterolemia, and stroke [34,35]. It reduces CV mortality in the healthy population [34]. It is also used both independently and as an ingredient in food products in therapy aimed at weight reduction and lowering waist circumference [36]. Dietary fiber is part of plant material and can be water-soluble or -insoluble [35]. It consists of plant matter, including carbohydrates and lignin, which are resistant to digestion and polysaccharides, oligo-saccharides, cellulose, starches, lipids, proteins, minerals, and tannins [35,37]. Vegetables, fruits, whole grains, potatoes, nuts, tubers, and legumes are the main source [37,38]. Water-insoluble dietary fiber has a beneficial effect on intestinal peristalsis [35]. Studies have shown that regular consumption of soluble fiber, whose sources include oat bran, oats, beans, and flax seeds, reduces blood LDL cholesterol levels [37]. Water-insoluble fibers such as wheat bran, cereals, bread, fruit and vegetable peels, nuts, seeds, rice, and pasta provide bulk to the stool and move it smoothly through the digestive tract, facilitating bowel movements and relieving constipation [37]. Regular consumption of soluble fiber, including beta-glucans from oats and barley, reduces the risk factor for cardiovascular disease. Additionally, those products regulate blood sugar levels, lowering glucose and insulin levels in people with diabetes, and may also reduce the risk of developing diabetes [37]. Dietary fiber, by influencing satiety, may contribute to improving individual parameters of body composition. In a study conducted by Barrett et al., besides the beneficial effect of dietary fiber on the lipid profile of the subjects, the authors also observed an inverse relationship between the consumption of whole-grain fiber and grains and the body mass index as well as waist circumference [39]. 

A strong relationship has been observed between dietary fiber intake and the risk of coronary heart disease. Wang et al. showed that higher fiber intake is associated with a 20–40% reduction of the risk of CAD [40,41]. Nweze et al. indicated in their paper that consuming oatmeal for a period of six weeks lowers systolic and diastolic blood pressure in hypertensive and hyperinsulinemic individuals [37]. Soliman’s research also suggests incorporating dietary fiber into statin therapy to increase treatment efficacy, reduce the recommended drug dose, and improve the patient’s health [42]. The recommended daily intake of dietary fiber as a food ingredient is 30–40 g, including 38 g for men and 25 g for women aged 19–50 years, and 31 g for men and 21 g for women aged >50 years [35,42]. According to the American Dietetic Association, the recommended intake is 25–30 g/day [38]. However, many studies point to an insufficient intake of dietary fiber. The median fiber intake among adults is 18.2 g in Australia and about 15–25 g in Scandinavian countries [43]. The estimated daily intake of dietary fiber is about 12 g for women and 24 g for men in France, Japan, Germany, the United Kingdom, Italy, and the United States [44]. As shown by Kolodziejczyk and Michniewicz, the intake of dietary fiber is also below the norm in Poland, with an average of 17 g per day [44]. In contrast, African countries and India have the highest dietary fiber intake in the world, with higher average intake in rural vs. urban areas [43]. Optimal or increased fiber intake is impossible for the group particularly at risk for cardiovascular diseases—elderly individuals, whose gastrointestinal issues necessitate a low-fiber diet. However, this type of diet exacerbates glycemic parameters and promotes the rapid absorption of lipids in the intestinal lumen [45,46].

### 3.5. Probiotics

Probiotics, which are nonpathogenic microorganisms, mainly include Lactobacillus, Lactococcus, Bacillus, Enterococcus, and Saccharomyces yeast [47]. Their sources include fermented dairy products, such as kefir, fermented milk, and yogurt, as well as plant-based products, such as oats, olives, pickled cucumbers, and sauerkraut [48,49,50]. Probiotics not only are involved in maintaining the homeostasis of the intestinal microflora but also support the treatment of CAD and improvement of certain components of metabolic syndrome such as obesity, hypertension, and glucose metabolism [51,52]. Probiotic bacteria reduce inflammation in men with CAD and the risk of preeclampsia. As reported in a literature review by Chen X et al., many clinical studies have shown that normal gut microbiota has beneficial effects on the cardiovascular system, protects endothelial function, and contributes to blood pressure regulation [50]. Endothelial dysfunction is considered a risk factor for cardiovascular diseases and appears during the development of hypertension, coronary heart disease, atherosclerosis, or stroke [53]. Endothelial function includes the control of platelet aggregation and blood hemostasis, regulating the antithrombotic–prothrombotic balance. Changes in the intestinal microbiota may impair endothelial function, contributing to a reduction in the vasodilatory response. However, probiotics can improve endothelial function and reduce the level of low-density lipoproteins, total cholesterol, and triglycerides.

Kefir, which is a natural probiotic, shows a blood-pressure-lowering effect [47]. An equally positive effect on blood pressure is attributed to fermented milk, which reduces arterial stiffness in hypertensive patients due to its content of Lactobacillus helveticus [47]. Yogurt lowers LDL cholesterol levels [47]. At the same time, it is observed that gut microflora imbalance is most common among patients with heart failure, thrombosis, and hypertension [50]. Costanza et al. showed that consumption of probiotics by patients with chronic systolic heart failure resulted in, among other things, reduced blood cholesterol and uric acid levels [54]. Fermented oats, olives, cabbage, and pickled cucumbers are rich in Lactobacillus plantarum bacteria, which play a trophic role in the intestines, ensuring biological properties and continuity of the gastrointestinal epithelium [48]. They increase blood flow to the large intestine, stimulating the motility of the digestive tract, which enhances the immune response by stimulating the production of antibodies (IgA) and reduces the intestinal microflora destroyed by gastrointestinal disorders or the use of antibiotics [47]. The number of living cells in probiotic food cannot be less than 106 cells in 1 mL or 1 g of the product for single consumption by a person. The therapeutic dose is 108–109 cells in 1 mL or 1 g of the product [47]. Given the above-described effects of probiotics, their use in CVD can be considered therapeutic.

### 3.6. Vitamins C and E

Cardiometabolic disorders most often progress with chronic increased oxidative stress. Reactive oxygen species contribute to vascular damage, thereby increasing the risk of thrombus formation [55,56]. Thus, oxidative stress predisposes one to atherosclerosis, coronary artery disease, and hypertension [56,57]. Foods rich in vitamins C and E, which primarily include fruits and vegetables, are nutritional antidotes to oxidative stress. The World Health Organization recommends that the daily intake of vegetables and fruits in the diet should be 400 g, noting that these products should be part of the five meals consumed during the day [58]. Authors of cohort studies clearly point to a negative correlation between the amount of vegetables consumed and CV risk [59]. As reported in the latest available literature, implementation of these recommendations is still insufficient.

Among myocardial infraction patients, 23.9% consume 1–2 servings of vegetables per day, while the remaining percentage consumes them at a lower frequency [60]. Nihat Küçük et al., who demonstrated the inadequate implementation of the WHO targets for the frequency of vegetable and fruit consumption among different social groups, suggest that policies are needed to support the possibility of purchasing these products in groups showing insufficient consumption [61]. Vitamin C reduces inflammatory markers, lowers blood pressure, and promotes nitric oxide secretion [62]. Valuable sources of vitamin C include parsley, bell peppers, sea buckthorn berries, and rosehips. In turn, vitamin E promotes the uptake of lipoperoxyl radicals, modulation of clotting processes, and reduction of oxidative stress, thereby preventing atherosclerosis and thrombus formation [63,64]. Published research indicates that consuming the recommended amount of vegetables and fruit provides vitamin C in the amount of 200–300 mg [65]. Sources of vitamin E include vegetable sprouts and vegetable oils, including olive oil [56]. The combined intake of products abundant in these two vitamins should provide additional benefits, since tocopherols and tocotrienols, after exerting their anti-inflammatory effects, are reduced by vitamin C and again show protective effects [62]. Therefore, it is beneficial to add vegetable oils to salads and raw vegetable salads. 

### 3.7. Vitamin B9 and B12

When it comes to cardiac prevention, special attention is paid to the benefits of consuming products that are sources of vitamins B9 and B12. Folic acid, which is found in the highest amounts in spinach, cabbage, broccoli, parsley, and beans, has a number of functions in the processes of cell division and synthesis of nucleic acids. Its intake is associated with lower blood pressure and a reduced risk of stroke [66,67,68]. Improvement of endothelial function, whose disorders contribute to the development of atherosclerotic processes, is the key benefit of vitamin B9 for patients with vascular disease [67]. However, it must be admitted that the bioavailability of vitamin B9 can be limited, but it can be increased by thermal treatment [69]. This improvement is possible due to the oxidative-stress-lowering effects as a result of reducing homocysteine levels in the vessels [58]. Vitamin B12, which is derived from offal and meat, is also needed to reduce homocysteine levels. Its deficiency leads to elevated lipid levels and the risk of obesity, which in turn increases the risk of CVD [70]. Individuals at risk of vitamin B12 deficiency include the elderly, those with gastric disorders, and vegetarians, for whom supplementation is necessary [71].

### 3.8. Arginine

Turkey meat and buckwheat groats are dietary products that provide the highest levels of arginine and at the same time (in the case of meat) do not contain ingredients with questionable effects on the lipid profile. Arginine is a nitrogen-rich amino acid used by the body to synthesize nitric oxide, a substance that improves blood flow and microcirculation [72]. Studies have shown that arginine intake is associated with reduced blood pressure, minimized CV risk factors and glycemic disorders, and lowered overall CV risk [73,74]. Moreover, the presumed impact of L-arginine supplementation as a promoter of nitric oxide production is considered to have a positive effect in preventing and treating metabolic syndrome and its phenotypes [75]. However, further research on the potential impact of arginine on metabolic disorders is necessary, as scientists have not assessed these relationships in relation to its blood concentration but only its dietary intake. This may be inaccurate due to individual differences in digestion and absorption of nutrients among study participants [75].

### 3.9. Allicin

Garlic is most often used as a flavoring agent in dishes due to its content of aromatic compounds, including allicin. This aromatic compound has broad cardioprotective effects, so this vegetable should be considered in the context of cardiometabolic prevention [76]. Garlic has been proven to reduce blood pressure, LDL cholesterol, total cholesterol, and triglycerides. It also helps reduce free radicals [76]. It is a recommended dietary product for atherosclerotic patients [77]. Black garlic, which is obtained by fermenting garlic and subjecting it to the Maillard reaction, also has cardioprotective properties [78]. After microbial fermentation processes, it shows stronger antioxidant effects, as well as relaxing effects in the arteries, preventing myocardial ischemia [78]. The beneficial effects of garlic should also be attributed to its influence on abdominal obesity and glucose levels. In four clinical trials conducted among individuals diagnosed with diabetes, it was observed that the use of garlic resulted in a reduction in blood glucose levels compared to the control group [79]. However, attention should be paid to the adverse effects that garlic consumption can cause, including interactions with anticoagulant and glucose-regulating medications. Therefore, its intake should be moderate, and issuing recommendations regarding its consumption could provide additional benefits [79].

### 3.10. Polyphenols

Polyphenols are natural antioxidants and beneficial components of fruits, vegetables, and, especially, honey [80,81,82]. They support the activity of endogenous components that eliminate free radicals, such as glutathione peroxidase, superoxide dismutase, and catalase [83]. Furthermore, they have also been shown to improve glucose tolerance and the lipid profile by lowering very-low-density lipoprotein triglycerides [82]. Honey consumption is recommended owing to its potential effects on vasomotor and endothelial function, which may help normalize blood pressure [82]. Although in vivo and in vitro studies on the efficacy of honey therapy are still underway, scientists are already concluding that the product should be used as a natural therapeutic ingredient for CVD [82]. However, it is worth mentioning the potential interactions of polyphenols with other nutrients such as starch, casein, and fats, which may limit their absorption and reduce their antioxidant potential [83,84].

### 3.11. Lycopene

Tomatoes, and especially tomato peel, are valued for their high content of lycopene, a carotenoid with the strongest anti-inflammatory properties, inhibiting the progression of cardiovascular disease and also preventing platelet aggregation, thereby reducing the risk of blood clot formation [85,86]. Tomatoes and tomato-containing products (sauces, purees, passatas) are recommended for individuals with atherosclerosis and a tendency to develop thromboembolic disease [87]. In addition to their high lycopene content, tomatoes are also a source of potassium, which is involved in regulating cardiac muscle function [72]. The deficiency increases the risk of heart disease [73]. The bioavailability of lycopene in food products can be increase by boiling or consuming tomatoes with olive oil [88]. The relationship between the absorption of lycopene and the presence of a fat additive is believed to be due to its lipophilic properties. Although studies indicate its poor solubility in edible fats, they emphasize that the total daily intake of fats may significantly affect the absorption of lycopene [89]. The panel of the European Food Safety Authority, based on clinical evidence involving humans, determined the permissible daily intake of lycopene at the level of 0.5 mg/kg of body weight per day [90].

A summary of cardiometabolic preventive compounds is presented in Table 1.

## 4. Food with Limited Salt and Sugar: The Importance of Harmful Food Compounds Elimination

Dietary salt restriction is particularly important in the prevention of and dietary therapy for hypertension as a metabolic syndrome component. According to the World Health Organization, the recommended daily salt intake for a healthy person should not exceed 5 g, and its significant minimization has cardiovascular benefits [23,91,92]. Limiting daily salt intake also has a beneficial effect on alleviating the clinical symptoms of heart failure [92]. In order to meet the needs in terms of combating hypertension, food manufacturers are developing food products with low sodium chloride content [93]. Meanwhile, according to research, salt intake is still too high, which, according to WHO data, is attributed to excessive consumption of highly processed foods [94]. In the United States alone, the average salt intake among people over the age of 25 years is 8.73 g [95]. Researchers indicate that the problem of too much salt in the diet already affects young Americans and Europeans, and they see the solution to this problem in effective public education [96,97].

Consumption of simple sugars predisposing one to hyperglycemia increases vascular inflammation and CV risk [98]. Sweetened beverages and foods containing simple sugars are considered harmful [99]. In order to optimize the lipid profile and reduce the risk of atherosclerotic cardiovascular disease, it is recommended to limit the consumption of simple sugars to 10% of total energy [24]. Studies indicate that sugar has an addictive effect, which likely contributes to its frequent consumption [100]. According to a report on the US population, bakery products are the most frequently consumed goods containing simple sugars [101]. Due to the excess consumption of sugar, the WHO recommends regulations, such as additional taxation of products containing sugars [101]. Meanwhile, products low in simple sugars, or with natural noncarbohydrate sweeteners, are available on the food market. Of particular note are products with added xylitol, such as baked goods and drinks. This ingredient has proven anti-inflammatory effects, which prevents bone demineralization and is a natural prebiotic [101].

## 5. CVD Dietary Therapy and Prevention Strategy for Individuals with Metabolic Syndrome: Nutritional Patterns and Practical Implementation Aspects

For managing metabolic syndrome and cardiovascular diseases (CVD), the literature highlights the Mediterranean diet as the primary nutritional strategy for both preventive and therapeutic purposes [101,102]. This diet model emphasizes increased consumption of vegetables and fruits, legumes, fish, and white meat, along with oils, while limiting intake of low-fiber products, red meat, and saturated fats [103]. According to recommendations from the American College of Cardiology, the American Heart Association, and the European Society of Cardiology, key dietary elements for CVD prevention include reducing sugary drinks, processed meat, saturated-fat-rich products, and salt in favor of plant-based foods [104]. However, as reviewed by Castro-Barquero, S. et al., it is not solely the diet model and elimination of certain foods but rather small beneficial modifications that show health benefits [101]. Petersen K. et al. reached similar conclusions, suggesting individualized and realistic dietary adjustments based on the patient’s capabilities, with dietitians advising on appropriate ingredient choices in line with nutritional recommendations [105].

A practical approach to implementing nutritional recommendations and lifestyle modifications must consider barriers to healthy eating [104]. For children, the most beneficial strategy is to promote a nutrient-rich, varied diet that is tolerant of food neophobia. Promoting proper eating behaviors in schools by offering exclusively healthful meals could help reduce the development of metabolic disorders and their consequences among the youngest populations.

For adults, diets should be individualized according to health needs, while for older individuals who may be at risk of nutritional deficiencies due to age-related conditions, it is essential to ensure proper nutritional status [104]. Dietary therapy proposed by specialists should consider economic feasibility and be highly tolerant of the patient’s cultural dietary patterns [104].

The ethnic aspect also plays a crucial role in exacerbating health inequalities resulting from adverse social, economic, and environmental conditions. Virani et al. highlight the necessity of interventions targeting the social determinants of health, particularly among African Americans, who face the highest risk of death from atherosclerotic heart disease compared to other ethnic groups [106].

The funding of dietary therapy services is another critical aspect. Entities responsible for financing medical treatments, including nutritional therapy, should consider subsidies for proposed dietary strategies to make their implementation as realistic as possible. Additionally, collaboration between the government and associations issuing dietary recommendations in each country is beneficial for promoting products with limited unhealthy components [107].

## 6. The Importance of Physical Activity and Stress Management in the Context of Cardiometabolic Disorders

Lack of physical activity is a major factor contributing to disease burden and mortality [108]. According to available information, specific types of physical activity and their components, such as the number of steps and duration of moderate physical activity, contribute to improved cardiometabolic health [109]. This is associated with the induction of metabolic adaptations in various tissues, which can overlap to enhance overall metabolism. An example of this effect is increased lipid turnover throughout the body due to elevated lipid oxidation and the expression of proteins regulating lipolysis. Furthermore, physical activity enhances insulin sensitivity in skeletal muscles and adipose tissue by stimulating GLUT4, which can complement other tissues [110]. This is confirmed by a study conducted by Slaght et al., which examined the impact of physical activity on the cardiometabolic health of adolescents with type 2 diabetes. The results clearly indicate a significant positive effect of physical activity on the cardiometabolic health of the participants [111]. Stress is another important factor contributing to the risk of cardiometabolic diseases. Regardless of its duration, stress has been linked to a 40–60% increased risk of ischemic heart disease, arterial stiffness, and type 2 diabetes. Chronic stress, in particular, is noted for causing increased allostatic load and dysregulation of the neuroendocrine, cardiovascular, and immune systems. This dysregulation involves multiple systems, including the HPA (hypothalamus–pituitary–adrenal) axis and the SAM (sympathetic–adrenal–medullary) system. Their dysregulation leads to an intensified inflammatory state, contributing to higher mortality risk and cardiovascular diseases [112,113].

## 7. Conclusions

Based on the latest available literature, this review presents food ingredients important for preventing cardiometabolic disorders and in their dietary therapy. These include plant sterols, foods high in fiber, probiotics, foods rich in antioxidant vitamins, polyphenols, folic acid, arginine, and CoQ10. Therefore, particularly beneficial effects are attributed to whole-grain products, vegetables and fruits, fermented products, white meat, vegetable oils, fish, tomatoes, garlic, and nuts, as well as limiting the consumption of products containing salt and simple sugars. The above foods are particularly valued due to their beneficial effects on the following:

- Lipid metabolism;
- Myocardial function;
- Anti-inflammatory effect;
- Glucose metabolism;
- Blood circulation.

The information presented in this narrative review supports, with recent findings, the view that dietary therapy and lifestyle modification may have a beneficial effect on patients suffering from cardiometabolic disorders, pointing to food ingredients, physical activity, and stress management as pillars of the prevention and treatment of lifestyle diseases. 

Simultaneously, there is the need for further research on the impact of food components on the prevention and treatment of cardiometabolic diseases, with particular focus on the dosage and interactions of individual components.

## Figures and Tables

**Table 1:** Protective effect of food compounds.

Compounds	Food Products	Protective Effects	Literature
Plant sterols and stanols	Vegetable oils, bread, vegetables, cereals, nuts	Reduction of total cholesterol Increase in cholesterol excretion Regulation of VLDLs secretion	[14,15,16]
Fiber	Bran and whole grains, vegetables, and fruits	Reduction in the risk of CAD Lowering systolic and diastolic blood pressure Improvement in body composition parameters	[37,39]
Probiotic bacteria	Fermented dairy and pickled vegetable products	Reduction of inflammation Reducing the risk of preeclampsia Endothelial protection Regulation of blood pressure Reduction in low-density lipoproteins, total cholesterol, triglycerides, and uric acid levels Enhancing the immune response	[47,51,52,54]
Folic acid	Green vegetables	Lowering blood pressure Reduction of the risk of stroke	[66,67]
Vitamin B12	White meat	Improvement of endothelial function Reduction in homocysteine levels	[70]
Vitamin C	Vegetables, fruits	Reduction of oxidative stress Lowering blood pressure Modulation of coagulation processes Reduction in low-density lipoproteins, total cholesterol, triglycerides Increase in HDL cholesterol	[62,63]
Vitamin E	Vegetables, fruits	Reduction of oxidative stress Lowering blood pressure Modulation of coagulation processes Reduction in low-density lipoproteins, total cholesterol, triglycerides Increase in HDL cholesterol	[62,63]
PUFAs	Oil products, fish	Reduction in blood triglycerides and reduction in postprandial lipemia Reduction of CVD risk	[21,22,23,24]
Lycopene	Tomatoes, tomato puree, passatas	Prevention of platelet aggregation Regulation of cardiac muscle function	[72,89]
Arginine	Turkey meat and buckwheat	Lowering blood pressure Reduction in CV risk factors Reduction of glycemic disorders	[73,74,75]
CoQ10	Wheat sprouts, fatty fish, oils, and nuts	Stabilization of calcium channel function Reduction in low-density lipoproteins, total cholesterol Reduction of the risk of atrial fibrillation Endothelial function improvement	[29,30]
Allicin	Fresh and fermented garlic	Reduction in low-density lipoproteins, total cholesterol, triglycerides Antioxidant effect	[77,78,79]

## Data Availability

Not applicable.

## References

[1] Dyrcz D., Przywara-Chowaniec B. (2019). Ocena predyspozycji młodych dorosłych do wystąpienia chorób układu krążenia. Forum Med. Rodz..

[2] Główny Urząd Statystyczny Umieralność w I Półroczu 2021 r. 31.01.2022 r. Zgony Według Przyczyny—Dane Wstępne. Umieralność w I Półroczu 2021 Roku 31.01.2022 r. Zgony Według Przyczyny—Dane Wstępne. https://stat.gov.pl/obszary-tematyczne/ludnosc/statystyka-przyczyn-zgonow/umielalnosc-w-2021-roku-zgony-wedlug-przyczyn-dane-wstepne,10,3.html.

[3] Sedaghat Z., Khodakarim S., Nejadghaderi S.A., Sabour S. (2024). Association between metabolic syndrome and myocardial infarction among patients with excess body weight: A systematic review and meta-analysis. BMC Public Health.

[4] Li X., Zhai Y., Zhao J., He H., Li Y., Liu Y., Feng A., Li L., Huang T., Xu A. (2021). Impact of Metabolic Syndrome and It’s Components on Prognosis in Patients With Cardiovascular Diseases: A Meta-Analysis. Front. Cardiovasc. Med..

[5] Di Pietro P., Izzo C., Carrizzo A. (2023). Editorial: The role of metabolic syndrome and disorders in cardiovascular disease. Front. Endocrinol..

[6] Wang Z., Chen J., Zhu L., Jiao S., Chen Y., Sun Y. (2023). Metabolic disorders and risk of cardiovascular diseases: A two-sample mendelian randomization study. BMC Cardiovasc. Disord..

[7] Guembe M.J., Fernandez-Lazaro C.I., Sayon-Orea C., Toledo E., Moreno-Iribas C. (2020). RIVANA Study Investigators. Risk for cardiovascular disease associated with metabolic syndrome and its components: A 13-year prospective study in the RIVANA cohort. Cardiovasc. Diabetol..

[8] Barkas F., Nomikos T., Liberopoulos E., Panagiotakos D. (2020). Diet and Cardiovascular Disease Risk Among Individuals with Familial Hypercholesterolemia: Systematic Review and Meta-Analysis. Nutrients.

[9] Zhang Y.B., Pan X.F., Chen J., Cao A., Xia L., Zhang Y., Wang J., Li H., Liu G., Pan A. (2021). Combined lifestyle factors, all-cause mortality and cardiovascular disease: A systematic review and meta-analysis of prospective cohort studies. J. Epidemiol. Community Health.

[10] Catapano A.L., Graham I., De Backer G., Wiklund O., Chapman M.J., Drexel H., Hoes A.W., Jennings C.S., Landmesser U., Pedersen T.R. (2016). 2016 ESC/EAS Guidelines for the Management of Dyslipidaemias. Kardiol. Pol..

[11] Topolska K., Florkiewicz A., Filipiak-Florkiewicz A. (2021). Functional Food-Consumer Motivations and Expectations. Int. J. Environ. Res. Public Health.

[12] Cicero A.F.G., Fogacci F., Stoian A.P., Vrablik M., Al Rasadi K., Banach M., Toth P.P., Rizzo M. (2021). Nutraceuticals in the Management of Dyslipidemia: Which, When, and for Whom? Could Nutraceuticals Help Low-Risk Individuals with Non-optimal Lipid Levels?. Curr. Atheroscler. Rep..

[13] Kaur R., Myrie S.B. (2020). Association of Dietary Phytosterols with Cardiovascular Disease Biomarkers in Humans. Lipids.

[14] Weingartner O., Patel S.B., Lutjohann D. (2020). It’s time to personalize and optimize lipid-lowering therapy. Eur. Heart J..

[15] Feng S., Belwal T., Li L., Limwachiranon J., Liu X., Luo Z. (2020). Phytosterols and their derivatives: Potential health-promoting uses against lipid metabolism and associated diseases, mechanism, and safety issues. Compr. Rev. Food Sci. Food Saf..

[16] Cedó L., Farràs M., Lee-Rueckert M., Escolà-Gil J.C. (2019). Molecular Insights into the Mechanisms Underlying the Cholesterol- Lowering Effects of Phytosterols. Curr. Med. Chem..

[17] Wożakowska-Kapłon B., Salwa P. (2016). Monakolina—Pomost między prozdrowotną modyfikacją diety a farmakoterapią hipercholesterolemii. Folia Cardiol..

[18] Poli A., Marangoni F., Corsini A., Manzato E., Marrocco W., Martini D., Medea G., Visioli F. (2021). Phytosterols, Cholesterol Control, and Cardiovascular Disease. Nutrients.

[19] Wang L., Feng L., Prabahar K., Hernández-Wolters B., Wang Z. (2024). The effect of phytosterol supplementation on lipid profile: A critical umbrella review of interventional meta-analyses. Phytother. Res..

[20] Sanllorente A., Lassale C., Soria-Florido M.T., Castañer O., Fitó M., Hernáez Á. (2021). Modification of High-Density Lipoprotein Functions by Diet and Other Lifestyle Changes: A Systematic Review of Randomized Controlled Trials. J. Clin. Med..

[21] Swora-Cwynar E., Wrotecki F., Dobrowolska A. (2022). Stan wiedzy lekarzy podstawowej opieki zdrowotnej na temat zasad żywienia chorych z zespołem metabolicznym. Forum Zaburzen Metab..

[22] Colussi G., Catena C., Novello M., Bertin N., Sechi L.A. (2017). Impact of omega-3 polyunsaturated fatty acids on vascular function and blood pressure: Relevance for cardiovascular outcomes. Nutr. Metab. Cardiovasc. Dis..

[23] Granato D., Barba F.J., Bursać Kovačević D., Lorenzo J.M., Cruz A.G., Putnik P. (2020). Functional Foods: Product Development, Technological Trends, Efficacy Testing, and Safety. Annu. Rev. Food Sci. Technol..

[24] Sikand G., Severson T. (2020). Top 10 dietary strategies for atherosclerotic cardiovascular risk reduction. Am. J. Prev. Cardiol..

[25] Shahidi F., Ambigaipalan P. (2018). Omega-3 Polyunsaturated Fatty Acids and Their Health Benefits. Annu. Rev. Food Sci. Technol..

[26] Khandouzi N., Zahedmehr A., Nasrollahzadeh J. (2020). Effects of canola or olive oil on plasma lipids, lipoprotein-associated phospholipase A_2_ and inflammatory cytokines in patients referred for coronary angiography. Lipids Health Dis..

[27] Smith A.D., Jernerén F., Refsum H. (2021). ω-3 fatty acids and their interactions. Am. J. Clin. Nutr..

[28] Lange K.W., Nakamura Y., Gosslau A.M., Li S. (2019). Are there serious adverse effects of omega-3 polyunsaturated fatty acid supplements?. J. Food Bioact..

[29] Dludla P.V., Nyambuya T.M., Orlando P., Silvestri S., Mxinwa V., Mokgalaboni K., Nkambule B.B., Louw J., Muller C.J.F., Tiano L. (2020). The impact of coenzyme Q10 on metabolic and cardiovascular disease profiles in diabetic patients: A systematic review and meta-analysis of randomized controlled trials. Endocrinol. Diabetes Metab..

[30] Chow S.L., Bozkurt B., Baker W.L., Bleske B.E., Breathett K., Fonarow G.C., Greenberg B., Khazanie P., Leclerc J., Morris A.A. (2023). American Heart Association Clinical Pharmacology Committee and Heart Failure and Transplantation Committee of the Council on Clinical Cardiology; Council on Epidemiology and Prevention; and Council on Cardiovascular and Stroke Nursing. Complementary and Alternative Medicines in the Management of Heart Failure: A Scientific Statement From the American Heart Association. Circulation.

[31] Sue-Ling C.B., Abel W.M., Sue-Ling K. (2022). Coenzyme Q10 as Adjunctive Therapy for Cardiovascular Disease and Hypertension: A Systematic Review. J. Nutr..

[32] Liu Z., Tian Z., Zhao D. (2022). Effects of Coenzyme Q10 Supplementation on Lipid Profiles in Adults: A Meta-analysis of Randomized Controlled Trials. J. Clin. Endocrinol. Metab..

[33] Dai S., Tian Z., Zhao D., Liang Y., Zhong Z., Xu Y., Hou S., Yang Y. (2024). The Association between the Diversity of Coenzyme Q10 Intake from Dietary Sources and the Risk of New-Onset Hypertension: A Nationwide Cohort Study. Nutrients.

[34] Merenkova S.P., Zinina O.V., Stuart M., Okuskhanova E.K., Androsova N.V. (2020). Effects of dietary fiber on human health: A Review. Hum. Sport. Med..

[35] Nweze C.C., Nebechukwu E.W., Bawa M.Y. (2021). Dietary fiber and risk of coronary heart diseases. GSCARR.

[36] Thomas M.S., Calle M., Fernandez M.L. (2023). Healthy plant-based diets improve dyslipidemias, insulin resistance, and inflammation in metabolic syndrome. A narrative review. Adv. Nutr..

[37] Nie Y., Luo F. (2021). Dietary Fiber: An Opportunity for a Global Control of Hyperlipidemia. Oxidative Med. Cell. Longev..

[38] Wang A.Y.M., Sea M.M.M., Ng K., Wang M., Chan I.H., Lam C.W., Sanderson J.E., Woo J. (2019). Dietary Fiber Intake, Myocardial Injury, and Major Adverse Cardiovascular Events Among End-Stage Kidney Disease Patients: A Prospective Cohort Study. Kidney Int. Rep..

[39] Barrett E.M., Batterham M.J., Beck E.J. (2020). Whole grain and cereal fibre intake in the Australian Health Survey: Associations to CVD risk factors. Public Health Nutr..

[40] Partula V., Deschasaux M., Druesne-Pecollo N., Latino-Martel P., Desmetz E., Chazelas E., Kesse-Guyot E., Julia C., Fezeu L.K., Galan P. (2020). Associations between consumption of dietary fibers and the risk of cardiovascular diseases, cancers, type 2 diabetes, and mortality in the prospective NutriNet-Santé cohort. Am. J. Clin. Nutr..

[41] Miller K.M. (2020). Review of whole grain and dietary fiber recommendations and intake levels in different countries. Nutr. Rev..

[42] Ramezani F., Pourghazi F., Eslami M., Gholami M., Mohammadian Khonsari N., Ejtahed H.S., Larijani B., Qorbani M. (2024). Dietary fiber intake and all-cause and cause-specific mortality: An updated systematic review and meta-analysis of prospective cohort studies. Clin. Nutr..

[43] Butnariu M., Sarac I. (2019). Functional Food. Int. J. Nutr..

[44] Kołodziejczyk P., Michniewicz J. (2018). Ziarno zbóż i produkty zbożowe jako źródła błonnika pokarmowego. Żywność Nauka Technol. Jakość.

[45] Saber A., Bayumi E. (2016). Age-Related Gastric Changes. J. Surg. Spec. Issue Gastrointest. Surg. Recent Trends.

[46] Chrzastek Z., Guligowska A., Sobczuk P., Kostka T. (2023). Dietary factors, risk of developing depression, and severity of its symptoms in older adults-A narrative review of current knowledge. Nutrition.

[47] Oniszczuk A., Oniszczuk T., Gancarz M., Szymańska J. (2021). Role of Gut Microbiota, Probiotics and Prebiotics in the Cardiovascular Diseases. Molecules.

[48] Dahiya D., Nigam P.S. (2023). Use of Characterized Microorganisms in Fermentation of Non-Dairy-Based Substrates to Produce Probiotic Food for Gut-Health and Nutrition. Fermentation.

[49] Pavlidou E., Fasoulas A., Mantzorou M., Giaginis C. (2022). Clinical Evidence on the Potential Beneficial Effects of Probiotics and Prebiotics in Cardiovascular Disease. Int. J. Mol. Sci..

[50] Chen X., Li H.Y., Hu X.M., Zhang Y., Zhang S.Y. (2019). Current understanding of gut microbiota alterations and related therapeutic intervention strategies in heart failure. Chin. Med. J..

[51] Vasquez E.C., Pereira T.M.C., Peotta V.A., Baldo M.P., Campos-Toimil M. (2019). Probiotics as Beneficial Dietary Supplements to Prevent and Treat Cardiovascular Diseases: Uncovering Their Impact on Oxidative Stress. Oxidative Med. Cell. Longev..

[52] Hsu C.N., Hou C.Y., Hsu W.H., Tain Y.L. (2021). Early-Life Origins of Metabolic Syndrome: Mechanisms and Preventive Aspects. Int. J. Mol. Sci..

[53] Rhee M., Lee J., Lee E.Y., Yoon K.H., Lee S.H. (2024). Lipid Variability Induces Endothelial Dysfunction by Increasing Inflammation and Oxidative Stress. Endocrinol. Metab..

[54] Costanza A.C., Moscavitch S.D., Faria H.C., Mesquita E.T. (2015). Probiotic therapy with Saccharomyces boulardii for heart failure patients: A randomized, double-blind, placebo-controlled pilot trial. Int. J. Cardiol..

[55] Shaito A., Aramouni K., Assaf R., Parenti A., Orekhov A., Yazbi A.E., Pintus G., Eid A.H. (2022). Oxidative Stress-Induced Endothelial Dysfunction in Cardiovascular Diseases. Front. Biosci..

[56] Senoner T., Dichtl W. (2019). Oxidative Stress in Cardiovascular Diseases: Still a Therapeutic Target?. Nutrients.

[57] Khosravi M., Poursaleh A., Ghasempour G., Farhad S., Najafi M. (2019). The effects of oxidative stress on the development of atherosclerosis. Biol. Chem..

[58] World Health Organization (2019). Healthy Diet. Regional Office for the Eastern Mediterranean. https://apps.who.int/iris/handle/10665/325828.

[59] Zhang H., Zeng Y., Yang H., Hu Y., Hu Y., Chen W., Ying Z., Sun Y., Qu Y., Li Q. (2021). Familial factors, diet, and risk of cardiovascular disease: A cohort analysis of the UK Biobank. Am. J. Clin. Nutr..

[60] Szczepańska E., Gacal M., Sokal A., Janota B., Kowalski O. (2023). Diet in Patients with Myocardial Infarction and Coexisting Type 2 Diabetes Mellitus. Int. J. Environ. Res. Public Health.

[61] Küçük N., Urak F., Bilgic A., Florkowski W.J., Kiani A.K., Özdemir F.N. (2023). Fruit and vegetable consumption across population segments: Evidence from a national household survey. J. Health Popul. Nutr..

[62] Boonthongkaew C., Tong-Un T., Kanpetta Y., Chaungchot N., Leelayuwat C., Leelayuwat N. (2021). Vitamin C supplementation improves blood pressure and oxidative stress after acute exercise in patients with poorly controlled type 2 diabetes mellitus: A randomized, placebo-controlled, cross-over study. Chin. J. Physiol..

[63] Fuentes E., Trostchansky A., Reguengo L.M., Junior M.R.M., Palomo I. (2021). Antiplatelet Effects of Bioactive Compounds Present in Tomato Pomace. Curr. Drug Targets.

[64] Garg A., Lee J.C. (2022). Vitamin E: Where Are We Now in Vascular Diseases?. Life.

[65] Padayatty S.J., Katz A., Wang Y. (2003). Vitamin C as an antioxidant: Evaluation of its role in disease prevention. J. Am. Coll. Nutr..

[66] Wang Y., Jin Y., Wang Y., Li L., Liao Y., Zhang Y., Yu D. (2019). The effect of folic acid in patients with cardiovascular disease: A systematic review and meta-analysis. Medicine.

[67] Zamani M., Rezaiian F., Saadati S., Naseri K., Ashtary-Larky D., Yousefi M., Golalipour E., Clark C.C.T., Rastgoo S., Asbaghi O. (2023). The effects of folic acid supplementation on endothelial function in adults: A systematic review and dose-response meta-analysis of randomized controlled trials. Nutr. J..

[68] Guarnizo-Poma M., Urrunaga-Pastor D., Montero-Suyo C., Lazaro-Alcantara H., Paico-Palacios S., Pantoja-Torres B., Benites-Zapata V.A., Insulin Resistance and Metabolic Syndrome Research Group (2018). Association between serum vitamin B12 levels and metabolic syndrome in a euthyroid population. Diabetes Metab. Syndr..

[69] Saini R.K., Nile S.H., Keum Y.S. (2016). Folates: Chemistry, analysis, occurrence, biofortification and bioavailability. Food Res. Int..

[70] Ashok T., Puttam H., Tarnate V.C.A., Jhaveri S., Avanthika C., Trejo Treviño A.G., Sl S., Ahmed N.T. (2021). Role of Vitamin B12 and Folate in Metabolic Syndrome. Cureus.

[71] Zhou L., Bai X., Wu B., Tan Y., Li M., Yang Q. (2024). Characterizing Vitamin B12 Deficiency in Neurology Outpatients: A Retrospective Observational Study. Clin. Neuropharmacol..

[72] Ma L., Hu L., Feng X., Wang S. (2018). Nitrate and Nitrite in Health and Disease. Aging Dis..

[73] An P., Wan S., Luo Y., Luo J., Zhang X., Zhou S., Xu T., He J., Mechanick J.I., Wu W.C. (2022). Micronutrient Supplementation to Reduce Cardiovascular Risk. J. Am. Coll. Cardiol..

[74] Gawrys J., Gajecki D., Szahidewicz-Krupska E., Doroszko A. (2020). Intraplatelet L-Arginine-Nitric Oxide Metabolic Pathway: From Discovery to Clinical Implications in Prevention and Treatment of Cardiovascular Disorders. Oxidative Med. Cell. Longev..

[75] Mirmiran P., Moghadam S.K., Bahadoran Z., Ghasemi A., Azizi F. (2017). Dietary L-Arginine Intakes and the Risk of Metabolic Syndrome: A 6-Year Follow-Up in Tehran Lipid and Glucose Study. Prev. Nutr. Food Sci..

[76] Imaizumi V.M., Laurindo L.F., Manzan B., Guiguer E.L., Oshiiwa M., Otoboni A.M.M.B., Araujo A.C., Tofano R.J., Barbalho S.M. (2022). Garlic: A systematic review of the effects on cardiovascular diseases. Crit. Rev. Food Sci. Nutr..

[77] Sobenin I.A., Myasoedova V.A., Iltchuk M.I., Zhang D.W., Orekhov A.N. (2019). Therapeutic effects of garlic in cardiovascular ath atherosclerotic disease. Chin. J. Nat. Med..

[78] Qiu Z., Zheng Z., Zhang B., Sun-Waterhouse D., Qiao X. (2020). Formation, nutritional value, and enhancement of characteristic components in black garlic: A review for maximizing the goodness to humans. Compr. Rev. Food Sci. Food Saf..

[79] Hosseini A., Hosseinzadeh H. (2015). A review on the effects of Allium sativum (Garlic) in metabolic syndrome. J. Endocrinol. Investig..

[80] Dryer-Beers E.R., Griffin J., Matthews P.M., Frost G.S. Higher dietary polyphenol intake is associated with lower blood inflammatory markers. J. Nutr..

[81] Bt Hj Idrus R., Sainik N.Q.A.V., Nordin A., Saim A.B., Sulaiman N. (2020). Cardioprotective Effects of Honey and Its Constituent: An Evidence-Based Review of Laboratory Studies and Clinical Trials. Int. J. Environ. Res. Public Health.

[82] Hashim K.N., Chin K.Y., Ahmad F. (2022). The Mechanism of Honey in Reversing. Metab. Syndr..

[83] Zhang H., Yu D., Sun J., Liu X., Jiang L., Guo H., Ren F. (2014). Interaction of plant phenols with food macronutrients: Characterisation and nutritional–physiological consequences. Nutr. Res. Rev..

[84] Tagliazucchi D., Helal A., Verzelloni E., Conte A. (2012). The type and concentration of milk increase the in vitro bioaccessibility of coffee chlorogenic acids. J. Agric. Food Chem..

[85] Vélez-Terreros P.Y., Romero-Estévez D., Navarrete H., Yánez-Jácome G.S. (2024). Nutritional Quality of Conventional, Organic, and Hydroponic Tomatoes Commercialized in Quito, Ecuador. Foods.

[86] Yamada S., Inaba M. (2021). Potassium Metabolism and Management in Patients with CKD. Nutrients.

[87] Gonçalves C., Abreu S. (2020). Sodium and Potassium Intake and Cardiovascular Disease in Older People: A Systematic Review. Nutrients.

[88] Perdomo F., Cabrera Fránquiz F., Cabrera J., Serra-Majem L. (2012). Influencia del procedimiento culinario sobre la biodisponibilidad del licopeno en el tomate. [Influence of cooking procedure on the bioavailability of lycopene in tomatoes]. Nutr. Hosp..

[89] Moran N.E., Erdman J.W., Clinton S.K. (2013). Complex interactions between dietary and genetic factors impact lycopene metabolism and distribution. Arch. Biochem. Biophys..

[90] Przybylska S., Tokarczyk G. (2022). Lycopene in the Prevention of Cardiovascular Diseases. Int. J. Mol. Sci..

[91] Report of the Formal Meeting of Member States to Conclude the Work on the Comprehensive Global Monitoring Framework, Including Indicators, and a Set of Voluntary Global Targets for the Prevention and Control of Communicable Diseases. Website. https://www.who.int/data/gho/indicator-metadata-registry/imr-details/3082.

[92] (2023). Mean Salt Intake in Adults Aged 25 Years and Older in the Americas, 1990–2019. ENLACE Data Portal. Pan American Health Organization. https://www.paho.org/en/enlace/salt-intake.

[93] Rosi A., Paolella G., Biasini B., Scazzina F. (2019). SINU Working Group on Nutritional Surveillance in Adolescents. Dietary habits of adolescents living in North America, Europe or Oceania: A review on fruit, vegetable and legume consumption, sodium intake, and adherence to the Mediterranean Diet. Nutr. Metab. Cardiovasc. Dis..

[94] Noroozi F., Fararouei M., Kojuri J., Ghahremani L., Ghodrati K. (2022). Salt Consumption and Blood Pressure in Rural Hypertensive Participants: A Community Filed Trial. Sci. World J..

[95] Luc K., Schramm-Luc A., Guzik T.J., Mikolajczyk T.P. (2019). Oxidative stress and inflammatory markers in prediabetes and diabetes. J. Physiol. Pharmacol..

[96] Yang B., Glenn A.J., Liu Q., Madsen T., Allison M.A., Shikany J.M., Manson J.E., Chan K.H.K., Wu W.C. (2022). Added Sugar, Sugar-Sweetened Beverages, and Artificially Sweetened Beverages and Risk of Cardiovascular Disease: Findings from the Women’s Health Initiative and a Network Meta-Analysis of Prospective Studies. Nutrients.

[97] Malik V.S., Hu F.B. (2019). Sugar-Sweetened Beverages and Cardiometabolic Health: An Update of the Evidence. Nutrients.

[98] Olszewski P.K., Wood E.L., Klockars A., Levine A.S. (2019). Excessive Consumption of Sugar: An Insatiable Drive for Reward. Curr. Nutr. Rep..

[99] Sebastian R.S., Enns C.W., Martin C.L., Goldman J.D., Moshfegh A.J. (2020). Sweet Foods Consumption by Adults in the U.S. What We Eat in America, NHANES 2015–2018. FSRG Diet. Data Briefs.

[100] Sahin A.W., Zannini E., Coffey A., Arendt E.K. (2019). Sugar reduction in bakery products: Current strategies and sourdough technology as a potential novel approach. Food Res. Int..

[101] Castro-Barquero S., Ruiz-León A.M., Sierra-Pérez M., Estruch R., Casas R. (2020). Dietary Strategies for Metabolic Syndrome: A Comprehensive Review. Nutrients.

[102] Chen W., Zhang S., Hu X., Chen F., Li D. (2023). A Review of Healthy Dietary Choices for Cardiovascular Disease: From Individual Nutrients and Foods to Dietary Patterns. Nutrients.

[103] Wang W., Liu Y., Li Y., Luo B., Lin Z., Chen K., Liu Y. (2023). Dietary patterns and cardiometabolic health: Clinical evidence and mechanism. MedComm.

[104] Belardo D., Michos E.D., Blankstein R., Blumenthal R.S., Ferdinand K.C., Hall K., Klatt K., Natajaran P., Ostfeld R.J., Reddy K. (2022). Practical, Evidence-Based Approaches to Nutritional Modifications to Reduce Atherosclerotic Cardiovascular Disease: An American Society For Preventive Cardiology Clinical Practice Statement. Am. J. Prev. Cardiol..

[105] Petersen K.S., Flock M.R., Richter C.K., Mukherjea R., Slavin J.L., Kris-Etherton P.M. (2017). Healthy Dietary Patterns for Preventing Cardiometabolic Disease: The Role of Plant-Based Foods and Animal Products. Curr. Dev. Nutr..

[106] Virani S.S., Alonso A., Aparicio H.J., Benjamin E.J., Bittencourt M.S., Callaway C.W., Carson A.P., Chamberlain A.M., Cheng S., Delling F.N. (2021). Heart Disease and Stroke Statistics-2021 Update: A Report From the American Heart Association. Circulation.

[107] Le Goff D., Aerts N., Odorico M., Guillou-Landreat M., Perraud G., Bastiaens H., Musinguzi G., Le Reste J.-Y., Barais M. (2023). Practical dietary interventions to prevent cardiovascular disease suitable for implementation in primary care: An ADAPTE-guided systematic review of international clinical guidelines. Int. J. Behav. Nutr. Phys. Act..

[108] Leskinen T., Stenholm S., Heinonen O.J., Pulakka A., Aalto V., Kivimaki M., Vahtera J. (2018). Change in physical activity and accumulation of cardiometabolic risk factors. Prev. Med..

[109] Meadley B., Perraton L., Smith K., Bonham M.P., Bowles K.A. (2022). Assessment of Cardiometabolic Health, Diet and Physical Activity in Helicopter Rescue Paramedics. Prehospital Emerg. Care.

[110] Ashcroft S.P., Stocks B., Egan B., Zierath J.R. (2024). Exercise induces tissue-specific adaptations to enhance cardiometabolic health. Cell Metab..

[111] Slaght J.L., Wicklow B.A., Dart A.B. (2021). Physical activity and cardiometabolic health in adolescents with type 2 diabetes: A cross-sectional study. BMJ Open Diabetes Res. Care.

[112] Lewis M.E., Volpert-Esmond H.I., Deen J.F., Modde E., Warne D. (2021). Stress and Cardiometabolic Disease Risk for Indigenous Populations throughout the Lifespan. Int. J. Environ. Res. Public Health.

[113] Bomhof-Roordink H., Seldenrijk A., Van Hout H.P., Van Marwijk H.W., Diamant M., Penninx B.W. (2015). Associations between life stress and subclinical cardiovascular disease are partly mediated by depressive and anxiety symptoms. J. Psychosom. Res..

